# Cryptococcal Phospholipase B1 Is Required for Intracellular Proliferation and Control of Titan Cell Morphology during Macrophage Infection

**DOI:** 10.1128/IAI.03104-14

**Published:** 2015-03-17

**Authors:** Robert J. Evans, Zhongming Li, William S. Hughes, Julianne T. Djordjevic, Kirsten Nielsen, Robin C. May

**Affiliations:** a Institute of Microbiology and Infection and School of Biosciences, University of Birmingham, Birmingham, United Kingdom; b Department of Microbiology, University of Minnesota, Minneapolis, Minnesota, USA; c University of Warwick, Coventry, United Kingdom; d Centre for Infectious Diseases and Microbiology, Westmead Millennium Institute, Westmead, New South Wales, Australia; e NIHR Surgical Reconstruction and Microbiology Research Centre, University Hospitals of Birmingham NHS Foundation Trust, Queen Elizabeth Hospital Birmingham, Birmingham, United Kingdom

## Abstract

Cryptococcus neoformans is an opportunistic fungal pathogen and a leading cause of fungal-infection-related fatalities, especially in immunocompromised hosts. Several virulence factors are known to play a major role in the pathogenesis of cryptococcal infections, including the enzyme phospholipase B1 (Plb1). Compared to other well-studied Cryptococcus neoformans virulence factors such as the polysaccharide capsule and melanin production, very little is known about the contribution of Plb1 to cryptococcal virulence. Phospholipase B1 is a phospholipid-modifying enzyme that has been implicated in multiple stages of cryptococcal pathogenesis, including initiation and persistence of pulmonary infection and dissemination to the central nervous system, but the underlying reason for these phenotypes remains unknown. Here we demonstrate that a Δ*plb1* knockout strain of C. neoformans has a profound defect in intracellular growth within host macrophages. This defect is due to a combination of a 50% decrease in proliferation and a 2-fold increase in cryptococcal killing within the phagosome. In addition, we show for the first time that the Δ*plb1* strain undergoes a morphological change during *in vitro* and *in vivo* intracellular infection, resulting in a subpopulation of very large titan cells, which may arise as a result of the attenuated mutant's inability to cope within the macrophage.

## INTRODUCTION

Cryptococcus neoformans is a pathogenic fungus that can cause severe and often fatal meningoencephalitis, especially in immunocompromised hosts. In recent years, C. neoformans has become a major emerging pathogen, due largely to the global HIV pandemic. In sub-Saharan Africa, for example, C. neoformans likely accounts for up to 44% of fatal HIV-related secondary infections (1). During infection, C. neoformans is known to interact closely with host macrophages (2) following inhalation of infectious spores from the environment into the lungs (3). Many virulence factors expressed by C. neoformans allow the fungus to evade phagocytosis by macrophages or to improve survival once phagocytosed (4).

One such virulence factor is phospholipase B1 (Plb1), a phospholipid-modifying enzyme with multiple enzymatic activities (5). In cryptococcal cells, a significant proportion of Plb1 enzymatic activity is cell wall associated, although secretion of Plb1 from Cryptococcus is known to occur *in vitro* (6). Plb1 is secreted following cleavage of a glycosylphosphatidylinositol (GPI) anchor motif, which anchors the enzyme to the cell wall (7, 8), and export of Plb1 to the cell wall is facilitated by Sec14-1, which is part of C. neoformans's protein export system (9). Plb1 has optimal activity under acidic conditions, at around pH 4.0 to 5.0 (conditions similar to that of the phagosome), and is active at 37°C (10).

In line with this putative role in pathogenesis, deletion of *PLB1* strongly attenuates virulence in C. neoformans (9, 11–13). Mice infected with a *PLB1* deletion C. neoformans strain (Δ*plb1*) show reduced fungal burden in the lungs (9). In addition, during murine infection Plb1 appears to be required for cryptococcal dissemination from the lungs into the bloodstream (12) and additionally may be involved in the translocation of cryptococcal cells across the blood-brain barrier into the brain (14).

The reason why the Δ*plb1* strain is attenuated during infection has not yet been fully elucidated. In this study, we sought to explore the role of Plb1 during macrophage infection. We report that Plb1 is critical for both proliferation and survival within the macrophage. In addition, we show that the Δ*plb1* strain responds to macrophage infection by significantly increasing both capsular diameter and overall cell size within the phagosome and in the murine lung. This morphology is reminiscent of previously reported titan cells (15–17) and suggests that control of cryptococcal cell size by Plb1 may play a vital role in the outcome of host macrophage infection.

## MATERIALS AND METHODS

### Ethics statement.

A total of 14 mice were handled in strict accordance with good animal practice, as defined by the relevant national and/or local animal welfare bodies. All animal work was approved by the University of Minnesota Institutional Animal Care and Use Committee (IACUC) under protocol no. 1308A30852.

### Strains, media, and cell lines.

The strains used in this work are listed in Table 1. Strains were stored long-term in MicroBank vials at −80°C. Strains were rescued onto yeast extract-peptone-dextrose (YPD) agar (YPD, 50 g/liter [Sigma-Aldrich]; 2% agar [Melford]) for 48 h at 25°C and then stored until needed at 4°C. Before experimentation, liquid cultures were grown from these stock plates in 2 ml YPD growth medium (50 g/liter) for 24 h at 25°C under constant rotation.

**TABLE 1:** Strains used in this study

Strain or genotype	Description	Reference
H99	C. neoformans serotype A mating type α; wild-type parent strain	
Δ*plb1*	*PLB1* gene knockout	10
Δ*plb1*::*PLB1*	Δ*plb1* strain with *PLB1* gene reconstituted	10
Δ*sec14*	*SEC14-1* gene knockout	13
Δ*sec14*::*SEC14*	Δ*sec14* strain with *SEC14-1* gene reconstituted	13

The J774 murine macrophage cell line was used for all *in vitro* infection assays. Cells were passaged in Dulbecco's modified Eagle medium (DMEM) culture media with serum (DMEM, low glucose, from Sigma-Aldrich; 10% fetal bovine serum [FBS] from Invitrogen; 1% 10,000 units penicillin–10 mg streptomycin from Sigma-Aldrich; 1% 200 mM l-glutamine from Sigma-Aldrich). Assays were performed with J774 cells between passages 4 and 15. Assays were performed in serum-free DMEM (DMEM [high glucose], 1% penicillin-streptomycin, 1% l-glutamine) unless otherwise stated.

### Intracellular proliferation assay.

The intracellular proliferation assay was performed as previously described (18). Briefly, 24 h before infection, 1 × 10^5^ J774 macrophages were seeded onto 24-well plastic plates (Greiner Bio One Cell Star) in 1 ml culture medium with serum and incubated for 24 h at 37°C and 5% CO_2_. Before infection, J774 cells were activated with 150 ng/ml phorbol 12-myristate 13-acetate in dimethyl sulfoxide (DMSO; Sigma) in 1 ml serum-free culture medium for 45 min. This medium was then replaced with serum-free medium alone. Simultaneously, overnight C. neoformans cultures grown at 25°C in YPD were washed three times in sterile 1× phosphate-buffered saline (PBS), quantified, and adjusted to 1 × 10^7^ cells per ml in PBS before opsonization for 1 h with 10 μg/ml anticapsular 18B7 antibody (a kind gift from Arturo Casadevall, Albert Einstein College of Medicine, New York, NY, USA). The activated J774 cells were then infected with 100 μl opsonized C. neoformans to give a multiplicity of infection (MOI) of 10 (e.g., 1 × 10^5^ J774 cells for 1 × 10^6^ C. neoformans cells) in unsupplemented serum-free DMEM and incubated for 2 h at 37°C, 5% CO_2_. After 2 h (time point 0), infected wells were washed at least 3 times with warm 1× PBS until all nonphagocytosed C. neoformans cells were removed from the wells.

For calculation of the intracellular proliferation rate, the number of internal cryptococci was determined at time points 0, 18, and 24 (time points 0, 0 + 18 h, and 0 + 24 h, respectively). At each time point, each well to be counted was washed three times with 1× PBS to remove any extracellular yeast. The macrophages in the well were then lysed in distilled water for 30 min. C. neoformans cells in the lysate were quantified at each time point with a cell-counting chamber, and the intracellular proliferation rate (IPR) was calculated by dividing the count at which the intracellular burden peaked by the count at time point 0. Viability testing of the recovered cells was achieved by diluting the lysate to give a concentration of 200 yeast cells per 100 μl and then plating onto YPD agar at 25°C for 48 h prior to counting CFU.

### Phagocytosis assay.

Twenty-four hours before assaying, J774 macrophages were seeded onto 13-mm glass coverslips (nitric acid treated) inside 2-cm^2^ 24-well plates at a concentration of 1 × 10^5^ cells per ml in 1 ml DMEM culture medium with serum and incubated at 37°C and 5% CO_2_. Activation and infection of the macrophages with opsonized C. neoformans cells followed the same protocol as described above for the intracellular proliferation assay. After 2 h of incubation, the cells were washed 3 times with 1× PBS to remove unphagocytosed cells and then fixed for 10 min with 250 μl 4% paraformaldehyde in PBS at 37°C. Following fixation, coverslips were washed with 1× PBS and sterile deionized water and mounted on glass slides using Mowiol mountant (100 mM Tris-HCl [pH 8.5], 9% Mowiol, 25% glycerol). Mounted coverslips were analyzed on a Nikon Ti-S inverted microscope fitted with a Plan apochromatic (APO) 60 × 1.40 differential interference contrast (DIC) oil immersion objective.

### Cell size assay.

J774 cells were plated into 24-well plates, activated, and infected with opsonized C. neoformans strains as described above for the IPR assay. Two hours postinfection, each well was washed at least 3 times with warm 1× PBS until all nonphagocytosed C. neoformans cells were removed from the wells, and 1 ml serum-free DMEM was then added to each well.

The 24-well plate was placed into an environmentally controlled stage (Okolabs) set to 30°C, 5% CO_2_. The cells were imaged using a Nikon TE2000 microscope fitted with a Digital Sight DS-Qi1MC camera and a Plan APO Ph1 20× dry objective. Images were recorded every 4 min for 20 h. Image acquisition and analysis was performed using the Nikon NIS Elements software package (Nikon).

Cell size was measured using the ellipse area tool, measuring from the center of each Cryptococcus cell to the edge, to obtain the diameter of each cell.

### *In vivo* titan cell and phagocytosis assay.

Cryptococcus neoformans cells were grown in YPD broth overnight. Cells were pelleted and resuspended in sterile phosphate-buffered saline at a concentration of 1 × 10^7^ cells/ml based on hemocytometer count. Groups of 6- to 8-week-old female A/J mice (Jackson Laboratory, Bar Harbor, ME) were anesthetized by intraperitoneal pentobarbital injection. Four to five mice per treatment were infected intranasally with 5 × 10^5^ cells in 50 μl PBS. At 3 days postinfection, mice were sacrificed by CO_2_ inhalation. Lungs were lavaged with 1.5 ml sterile PBS three times using a 18.5-gauge needle placed in the trachea. Cells in the lavage fluid were pelleted and fixed in 3.7% formaldehyde at room temperature for 30 min. Cells were washed once with PBS, and >500 cells per animal were analyzed for size by microscopy (AxioImager, Carl Zeiss, Inc.). Cell body sizes were measured, and cells were classified as small cells (<15 μm in cell body diameter) or titan cells (>15 μm in cell body diameter).

### Titan cell flow cytometry.

H99 was cultured in YPD broth overnight. Cells were pelleted, washed three times in PBS, and fixed in 3% paraformaldehyde at room temperature for 10 min. Bronchoalveolar lavage (BAL) samples were washed once in 0.05% sodium dodecyl sulfate (SDS) to lyse mammalian cells and then washed three times in sterile water. Cells were pelleted and fixed in 3% paraformaldehyde at room temperature for 10 min. Following fixation, samples were washed with PBS, stained with 0.5 μg/ml Hoechst (Sigma, St. Louis, MO) at room temperature for 10 min, washed again with PBS, and then resuspended in PBS. Approximately 10,000 cells were examined for cell size by forward scatter (FSC) and for DNA content by Hoechst staining using an LSRII flow cytometer with FACSDiva software (BD Biosciences, San Jose, CA). Graphs were generated using Flowjo software (Tree Star, Inc., Ashland, OR). The *t* test was used to analyze the differences in titan cell formation and phagocytosis, and *P* values of <0.05 were considered significant.

### Cell stress.

Overnight C. neoformans cultures in 2 ml YPD were grown at 25°C. Cultures were diluted 1:1,000 into 500 μl buffered YPD (pH 7.0, 15 mM HEPES) in plastic-bottomed 48-well plates (Greiner) with various concentrations of SDS (0, 0.01, 0.05, 0.1, 0.25, and 0.5 mM), of H_2_O_2_ (0, 0.125, 0.25, 0.5, 1, 3, 6, and 14 mM), or of NaCl (0, 125, 250, 500, 1,000 mM). The plate was then sealed with a breathable membrane, and growth curve assays were performed for 24 h at 30°C in a Fluostar Omega plate reader using a custom script that took 600-nm absorbance readings from the bottom of the plate every 30 min. Between reads, the plate was shaken at 200 rpm with a linear shaking pattern.

### Statistics.

Unless otherwise stated, all statistics were performed using Prism Graphpad software. For cell size measurement comparison between time points, a Mann-Whitney U test was performed (e.g., StrainX 0 h versus StrainX 18 h). Unless stated otherwise, a *P* value below 0.05 was taken as significant. Unless otherwise stated, all figures have error bars representing standard errors. Significance is shown on figures by asterisks as follows: *, *P* ≤ 0.05; **, *P* ≤ 0.01; ***, *P* ≤ 0.001; and ****, *P* ≤ 0.0001.

## RESULTS

### Plb1 deficiency leads to increased uptake of C. neoformans cells by J774 macrophages.

**FIG 1:**
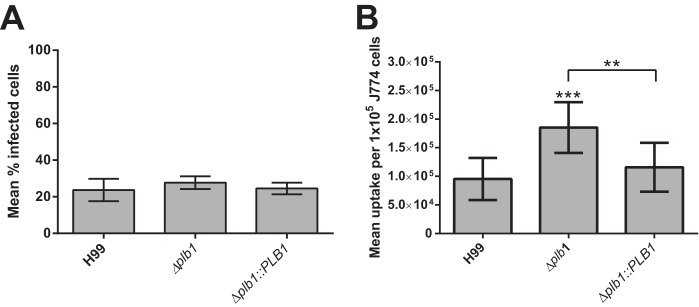
(A) Percentages of macrophages within a population that show C. neoformans infection following 2-h incubation with opsonized cryptococci at an MOI of 1:10 (*n =* 4). A minimum of 500 macrophages were counted for each condition. (Unpaired *t* test was performed, no significance.) (B) Total burden of infection within a fixed population of J774 cells (1 × 10^5^) following 2-h incubation with opsonized cryptococci at an MOI of 1:10 (*n =* 9). ***, H99 versus Δ*plb1* strain, *P* = 0.0003; **, Δ*plb1* versus Δ*plb1*::*PLB1* strains, *P* = 0.0038 (unpaired *t* test).

We first assessed to what extent the *PLB1* knockout strain, Δ*plb1* strain, was phagocytosed by macrophages in comparison to the wild-type parent strain H99 and a genetically reconstituted strain, Δ*plb1*::*PLB1* strain. We found that, following 2 h of infection, the percentage of J774 murine macrophages with at least one internalized C. neoformans cell did not differ significantly between wild-type strain H99, Δ*plb1* strain, and Δ*plb1*::*PLB1* reconstituted strain (Fig. 1A). Quantification of the fungal burden per macrophage, however, revealed a 2-fold increase in the number of Δ*plb1* cells phagocytosed per host cell (Fig. 1B, *P* = 0.0003). This suggests that Plb1 activity contributes significantly to the well-known antiphagocytic phenotype of cryptococci.

### Plb1 is required for intracellular proliferation of C. neoformans within J774 macrophages.

**FIG 2:**
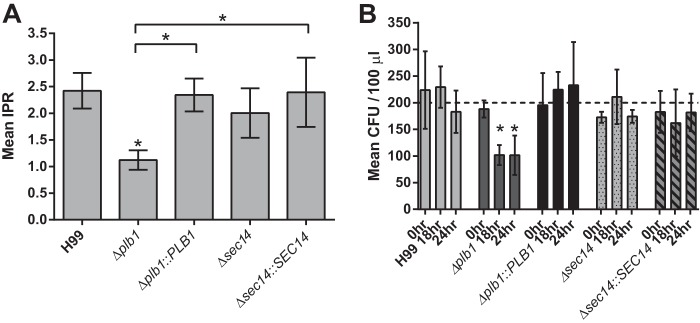
(A) Mean intracellular proliferation rate for each C. neoformans strain within murine J774 macrophages (*n =* 3). *, H99 versus Δ*plb1* strain, *P* = 0.023; *, Δ*plb1* versus Δ*plb1*::*PLB1* strains, *P* = 0.033; *, Δ*plb1* versus Δ*sec14*::*SEC14* strains, *P* = 0.0263 (one-way analysis of variance [ANOVA] plus Tukey posttest). (B) Viability of C. neoformans cells recovered from J774 macrophages following the IPR assay. Cells recovered following macrophage lysis were counted and diluted, giving an expected plated inoculum of 200 cells (*n =* 4). *, Δ*plb1* strain 0 h versus Δ*plb1* strain 18 h, *P* = 0.0422; *, Δ*plb1* strain 0 h versus Δ*plb1* strain 24 h, *P* = 0.0415 (two-way ANOVA plus Tukey posttest comparing time points for each strain).

*PLB1* knockout leads to a reduced fungal lung burden during murine infection; in addition, it abrogates dissemination to the brain (9, 14) and has previously been shown to reduce budding of C. neoformans within macrophages (11). To investigate this phenotype further, we performed an intracellular proliferation rate assay. We observed an intracellular proliferation defect for the Δ*plb1* strain versus the wild-type strain (IPR = 1.12 versus 2.42 for H99; *P* = 0.023), a defect that was fully restored in the Δ*plb1*::*PLB1* reconstituted strain (Fig. 2A). Interestingly, no intracellular proliferation defect was observed when we tested the Δ*sec14* strain (Fig. 2A). Since Sec14-1 is required for efficient secretion of Plb1 (9), it appears that intracellular, but not secreted, Plb1 is important for proliferation within host cells.

Due to the increased efficiency of Δ*plb1* cell uptake observed, we wondered whether the reduced proliferation we see for Δ*plb1* cells simply reflects initial overcrowding within the host macrophage. To test this, we reduced the uptake of Δ*plb1* cells by omitting the opsonization step before infection. Infecting with unopsonized Cryptococcus reduced uptake for all three strains (see Fig. S1Ci in the supplemental material: unopsonized Cryptococcus can still be phagocytosed by macrophage but at a lower rate [19, 20]) but did not alter the growth defect observed (see Fig. S1Cii in the supplemental material). To test a different method of opsonization, we also tried opsonizing with pooled serum instead of antibody. At the concentration used (5% pooled human serum), we did not see significant differences in uptake, compared to antibody opsonization (see Fig. S1Bi in the supplemental material), and the proliferation defect remained (see Fig. S1Bii in the supplemental material). Taken together, these data suggest that Plb1 plays a direct role in regulating intracellular proliferation in cryptococci regardless of the fungal intracellular burden.

### Plb1 contributes to C. neoformans cell survival during infection.

The low intracellular proliferation defect observed for Δ*plb1* could be due to slower Cryptococcus proliferation and/or increased intracellular killing by the macrophage. To address the second possibility, we plated C. neoformans cells recovered from macrophages at 18 and 24 h postinfection on YPD agar to enumerate CFU. No significant difference in CFU among any of the five strains was seen at time point 0 (2 h postinfection); however, at time point 18 (20 h postinfection), the viability of Δ*plb1* strain but not that of the Δ*sec14-1* strain had dropped significantly (*P* = 0.04) (Fig. 2B) compared to that of H99. Taken together, these data suggest that both growth and survival of the Δ*plb1* strain are impaired within the phagosome during macrophage infection.

### Plb1 deficiency does not significantly affect cryptococcal stress responses.

**FIG 3:**
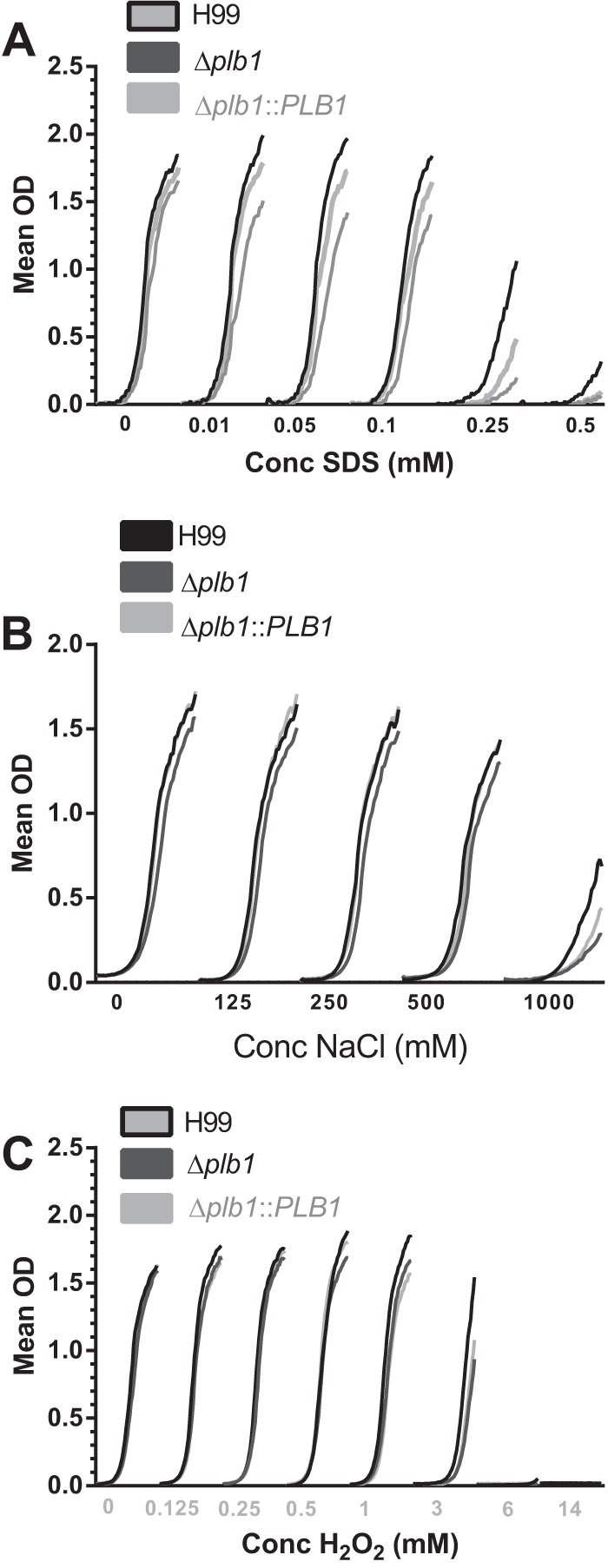
Overlaid growth curves with increasing concentrations of stress factors that partly mimic stresses in the phagosome. (A) SDS (membrane instability); (B) NaCl (osmotic shock); (C) H_2_O_2_ (reactive oxygen).

We hypothesized that low IPR in the macrophage may be a result of stressful conditions during macrophage infection—possibly because the Δ*plb1* strain is unable to control or resist cellular stresses within the macrophage. However, exposing the Δ*plb1* strain to a variety of stresses showed no significant difference from the wild type other than the previously reported susceptibility to SDS (9) (Fig. 3A and B).

### *PLB1* knockout leads to changes in cryptococcal cell body morphology.

**FIG 4:**
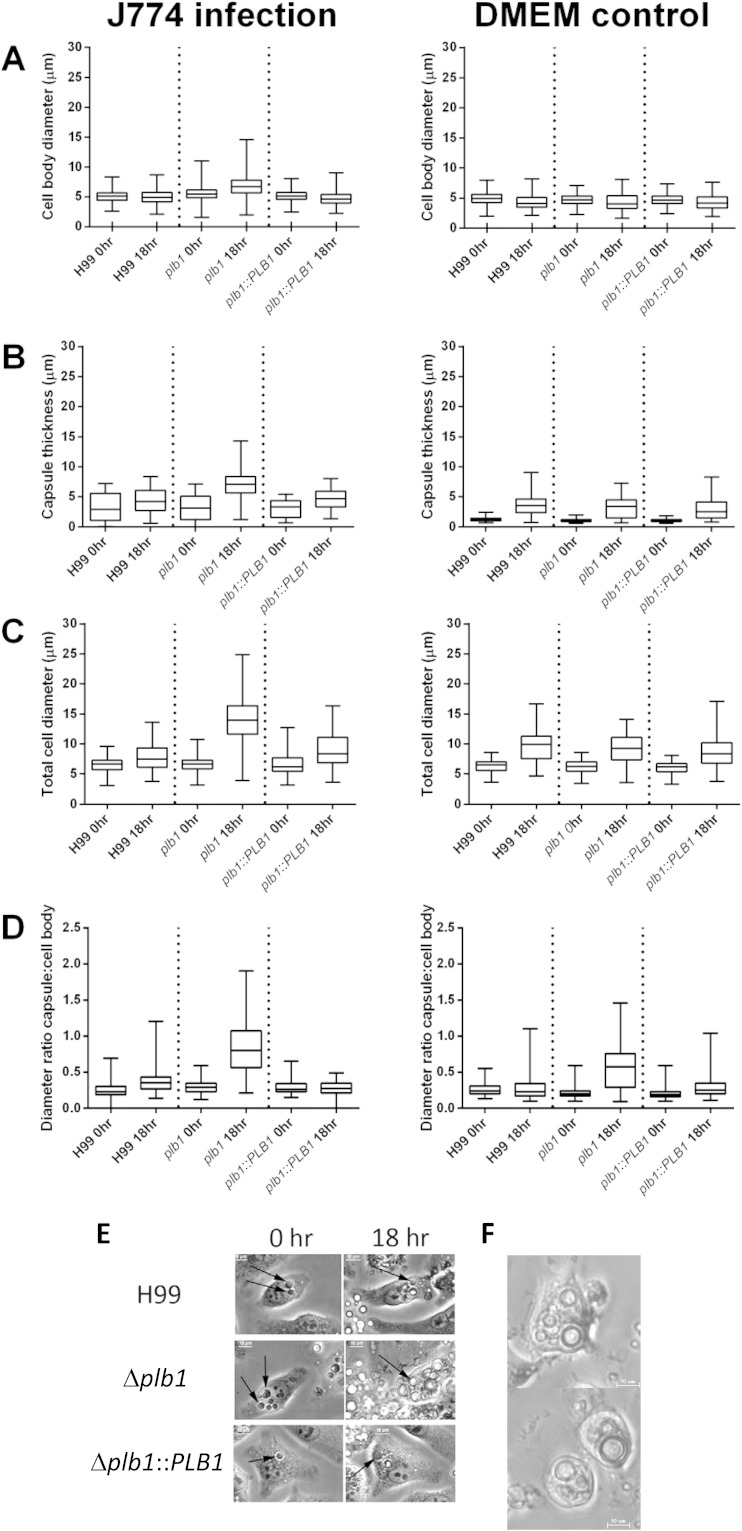
(A to D) C. neoformans cells were measured after either 18 h inside J774 macrophages or 18 h in serum-free DMEM. The top and bottom of each box mark the 75th and 25th percentiles, respectively, the bisecting line in the box marks the median, and the two whiskers mark the maximum and minimum of the range. Mann-Whitney U tests were performed for each time point pair. Full statistical results are given in Tables S1 and S2 in the supplemental material. (B) Capsule thickness was calculated by subtracting cell body diameter from total diameter including capsule. (D) The ratio of cell body to capsule was calculated by dividing cell body diameter by capsule thickness. (E) Still bright-field images from live-cell microscopy following the same macrophage over 18 h for H99, Δ*plb1*, and Δ*plb1*::*PLB1* strains. Black arrows indicate the location of single C. neoformans cells within the macrophage. (F) Still bright-field images from live-cell microscopy following viable Δ*plb1* strain-infected macrophages. Images were taken at 40 h postinfection; bar, 10 μm.

During the course of this work, we noticed that some Δ*plb1* cells showed a marked increase in cell body diameter within infected macrophages, a morphology that was not observed for H99 or the Δ*plb1*::*PLB1* strain (Fig. 4E and F). Quantification of this phenomenon (Fig. 4) showed that the diameter of both the cell body and the capsule of Δ*plb1* cells increased significantly during an 18-h macrophage infection (Fig. 4A to E). Interestingly, we observed a significant decrease in cell diameter for H99 and Δ*plb1*::*PLB1* cells over the same incubation period (see Table S1 in the supplemental material), which is probably due to active cell budding occurring within the macrophage, leading to a drop in mean diameter for the overall population. Extending the period of live cell imaging to 48 h, we found that the enlarged Δ*plb1* cells did not noticeably increase in size after 18 h postinfection.

**FIG 5:**
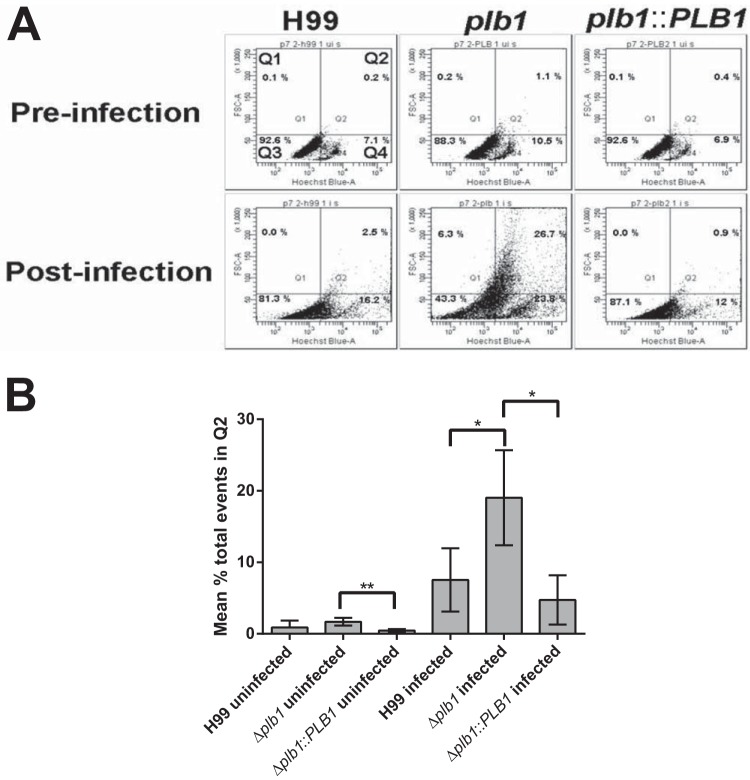
(A) Flow cytometric analysis of C. neoformans samples pre- and postincubation with J774 macrophages (18 h). Samples were stained with Hoechst to quantify DNA content; FSC-A (forward scatter area) indicates the relative cell size across the samples. Cells were gated based on size and nuclear content with cells in Q3 consistent with small cells in G_1_, cells in Q4 indicative of small cells in G_2_, and cells in Q2 indicative of polyploidy titan cells with both large size and increased DNA content. (B) Mean percentage of total events (10,000 collected) across 3 repeats occurring in the Q2 gate. A one-tailed *t* test was performed for H99 and Δ*plb1* strains as well as Δ*plb1*and Δ*plb1*::*PLB1* strains to determine if the increase in events within the Q2 gate observed was significant: *, H99-infected versus Δ*plb1* strain-infected cells, *P* = 0.034; *, Δ*plb1* strain-infected versus Δ*plb1*::*PLB1* strain-infected cells, *P* = 0.015; **, Δ*plb1* strain-uninfected versus Δ*plb1*::*PLB1* strain-uninfected cells, *P* = 0.001.

Changes in Cryptococcus cell size during *in vivo* infection have been previously documented, ultimately resulting in the production of very large titan cells (15–17, 21). To our knowledge, though, this is the first published observation of a similar (albeit less dramatic) process occurring within a macrophage (rather than extracellularly) and the first time that *PLB1* has been implicated in this pathway. Previous studies have reported that the size increase observed in titan cells is accompanied by increased nuclear content due to polyploidy (15, 16). In agreement with this, flow cytometric analysis of Δ*plb1* cells following macrophage infection demonstrated a positive correlation between cell size and DNA quantity (Fig. 5A), indicating increased nuclear content within these cells.

### Δ*plb1* cells have a higher rate of titan cell formation than do wild-type cells during *in vivo* murine infection.

**FIG 6:**
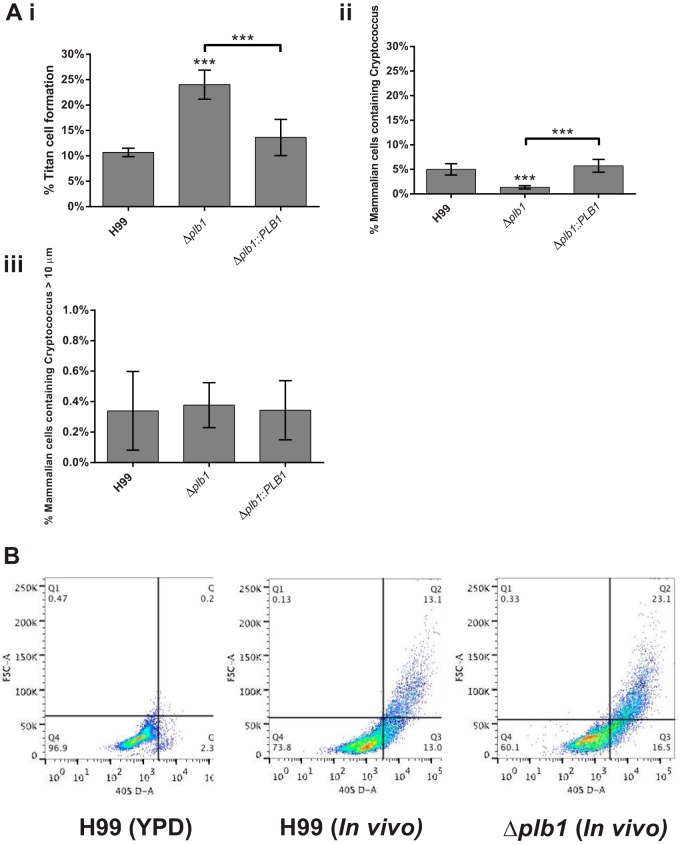
*In vivo* titan cell assay on Cryptococcus cells lavaged from mouse lungs 3 days postinfection. (Ai) A minimum of 500 cells per animal were analyzed to quantify cell body diameter, Cryptococcus cells >15 μm in diameter were considered titan cells (***, H99 versus Δ*plb1* strain, *P* = 0.0001; ***, Δ*plb1* versus Δ*plb1*::*PLB1* strains, *P* = 0.0018 [two-tailed unpaired *t* test]). (Aii) Host cells lavaged from the lungs were examined for phagocytosed Cryptococcus cells (***, H99 versus Δ*plb1* strain, *P* = 0.0001; ***, Δ*plb1* versus Δ*plb1*::*PLB1* strains, *P* = 0.0001 [two-tailed unpaired *t* test]). (Aiii) The cell body diameter of phagocytosed Cryptococcus cells within lavaged host macrophages was quantified, and data for phagocytosed cells >10 μm are given. (B) Bronchoalveolar lavage samples with Cryptococcus from H99 and Δ*plb1* strains were stained with Hoechst and analyzed for size and nuclear content. Cells were gated based on size and nuclear content with cells in Q3 consistent with small cells in G_1_, Q4 indicative of small cells in G_2_, and Q2 indicative of polyploidy titan cells with both large size and increased DNA content. (C) Bright-field images of lavaged cells from H99, Δ*plb1*, or Δ*plb1*::*PLB1* strain-infected lungs. Bar, 15 μm.

Our *in vitro* observation of Δ*plb1* cell enlargement was reminiscent of the titan cell morphology observed during *in vivo* murine pulmonary infection (15, 16, 21). To test whether this phenotype was recapitulated *in vivo*, we conducted pulmonary infections in mice and then quantified cryptococcal size following bronchoalveolar lavage (BAL) at 3 days postinfection. As with our *in vitro* data, *in vivo* infection with Δ*plb1* cells produced about 2.5 times more titan cells (cryptococci with a cell body diameter greater than 15 μm) than H99 or Δ*plb1*::*PLB1* cells (Fig. 6Ai). Flow cytometry of lavaged, Hoechst-stained Δ*plb1* titan cells indicated that these cells are polyploid, in agreement with previously published characterization of titan cells (15, 16) as well as our own *in vitro* observation of Δ*plb1* cells (Fig. 5A). Taken together, these findings suggest that the morphological changes that we observed in our Δ*plb1* cells are consistent with an increase in the production of titan cells by this strain both *in vitro* and *in vivo*.

### *In vivo* Δ*plb1* titan cell formation is not associated with phagocytosed cells.

In addition to quantifying overall titan cell formation in BAL samples, we also counted the Cryptococcus cells within host phagocytes following BAL and measured the cell body diameter of each Cryptococcus cell within each lavaged host cell.

We found that there were significantly fewer lavaged host cells containing Cryptococcus for Δ*plb1* strain-infected mice than for mice infected with H99 or Δ*plb1*::*PLB1* strain (Fig. 6Aii, H99 versus Δ*plb1*, *P* = 0.0001; Δ*plb1* versus Δ*plb1*::*PLB1*, *P* = 0.0018). Very few of these internalized Cryptococcus cells were above 10 μm in diameter, and those that were above 10 μm could be found in similar numbers for H99, Δ*plb1*, and Δ*plb1*::*PLB1* cells (Fig. 6Aiii). No cells greater than 15 μm were found inside phagocytes in either the control or the Δ*plb1* strain infections (data not shown). This leads us to conclude that *in vivo* Δ*plb1* titan cell formation potentially occurs initially within host cells (as we observe *in vitro*) but these large cryptococci are released from host cells either by host cell lysis *in vivo* or due to the physical process of lavage prior to the time point examined.

## DISCUSSION

During cryptococcal pathogenesis, C. neoformans interacts with professional phagocytes in the alveolar space (2), in the bloodstream, and in other tissues following dissemination. The outcome of this interaction appears to be vital for disease progression, as the macrophage provides a protective niche for replication (18) and a potential “Trojan horse” for dissemination to the central nervous system (22, 23). Together with previous studies, our findings strongly suggest that expression of Plb1 is critical in regulating cryptococcus-macrophage interactions.

First, we show that *PLB1* deletion leads to enhanced uptake by phagocytes *in vitro* (Fig. 1B). Since Plb1 is a GPI-linked, cell wall-associated protein (7), it is likely that loss of this enzyme affects the cryptococcal cell surface and, presumably, this change facilitates more-efficient binding of phagocyte receptors. Examination of different opsonization methods (see Fig. S1 in the supplemental material) suggests that increased uptake is due to a combination of antibody, complement, and nonopsonic ligands. Cryptococcal cell wall instability following *PLB1* deletion has been published previously (8), and thus this instability could lead to increased ligand exposure on the Cryptococcus cell surface. In light of these observations, a detailed chemical analysis of the cell surface composition in this strain would be of considerable future interest.

Following phagocytosis, we find that C. neoformans requires the expression of Plb1 to proliferate and survive normally within the macrophage (Fig. 2). This finding provides a mechanism to explain previous reports of reduced fungal burdens following infection with Δ*plb1* cells (9). Interestingly, however, the Δ*sec14-1* strain shows no such defect in intracellular proliferation. Since this strain shows strongly reduced Plb1 secretion (9), the most likely explanation is that the primary role of Plb1 in driving intracellular proliferation is in the metabolism of fungal phospholipids within the cryptococcal cell, rather than in acting directly upon host phospholipids. However, secreted Plb1 appears to have additional roles in virulence, since the Δ*sec14-1* strain is attenuated in animal models (9). It is tempting to speculate that the documented involvement of *SEC14* in regulating vomocytosis (9) may be one such role.

A striking morphological observation arising from our macrophage infection model was that Δ*plb1* cells showed a marked increase in both cell body diameter and capsule thickness during infection, with a maximum measured diameter (including capsule) of almost 25 μm (Fig. 4C). That this size change was not seen in the wild-type or Δ*plb1*::*PLB1* reconstituted strains suggests that this morphology results from the loss of Plb1. Previously reported titan cells grown *in vivo* range in diameter from 15 to 100 μm, excluding the capsule (15, 16). Therefore, the size increase that we observed is smaller than that of previously reported titan cells but significantly larger than the normal cryptococcal size range. Interestingly, previous reports of titan cells induced *in vitro* also noted that the cells tended to be smaller than those generated *in vivo* (16). Flow cytometry analysis of Δ*plb1* cells following macrophage infection (Fig. 5) indicates that the large cells show increased DNA content, consistent with the defining feature of titan cells (16). Thus, these cells may represent individual yeast cells that are “en route” to becoming Titan cells or have somehow been constrained by the macrophage, or it may be that the *in vitro* conditions do not provide the necessary stimulus to produce the largest titan cells.

From our *in vitro* data, we conclude that the Δ*plb1* strain forms large cells within macrophages, which are similar to the titan cell morphology seen *in vivo*. Titan cell formation has previously been induced *in vitro* using macrophage-conditioned media (16); however, titan cell formation induced inside an infected macrophage has to our knowledge not previously been reported. Although we have not identified the trigger for this morphology, it is evidently linked to Plb1 deficiency. The development of this morphology within the macrophage may be a response to increased stress experienced by the Δ*plb1* strain, as indicated by the decreased viability of this strain within the phagosome. Although we did not detect any significant difference stress tolerance for Δ*plb1* organisms *in vitro*, other than the previously published susceptibility to SDS (9), growth within the phagosome exposes cryptococci to a diverse repertoire of additional stresses, and it is likely that these complex conditions limit the growth of this mutant. One possible source of stress within the phagosome, which links our observed phenotypes, is the observation that in wild-type cryptococci, Plb1 concentrates at the neck of newly forming buds (24). This suggests that Plb1 may have a role in bud development, and thus it is conceivable that modification of membrane phospholipids by Plb1 during budding could be required for membrane curvature at the newly formed bud site or release of the daughter cell. The inability of the Δ*plb1* strain to properly bud would explain the proliferation defect observed and could generate sufficient cellular stress to reduce cryptococcal viability and trigger the development of titan-like cells.

A strong link between our *in vitro* observations and previously published *in vivo* development of titan cells is supported by our finding that the Δ*plb1* strain forms greater numbers of “classical” titan cells (i.e., cell body diameter above 15 μm and high nuclear content indicative of polyploidy) *in vivo* than do the wild-type and reconstituted strains (Fig. 6Ai). These data suggest that Plb1 activity is involved in the repression of titan cell formation during infection. In addition, the fact that the Δ*sec14-1* strain does not manifest similar cell size increases during *in vitro* infection (our unpublished data) indicates that intracellular rather than extracellular Plb1 activity regulates the titan cell morphology. Interestingly, titan cell development may be linked to phospholipid availability (25), suggesting a potential link to Plb1 activity. Titan cell formation *in vivo* has been reported to increase the pathogenicity of C. neoformans (16, 17); our conclusion that the Δ*plb1* strain has increased titan cell formation therefore seems at odds with the *in vitro* data presented in Fig. 1 and 2 showing that the Δ*plb1* strain is attenuated in macrophages. It is therefore possible that the large Δ*plb1* cells may not exhibit all the features of titan cells (for instance, in conferring “cross-protection” on nontitan cells in the same host). Alternatively, it may be that full pathogenicity requires only a minor titan cell population and hence, the enhanced titan cell frequency seen in the Δ*plb1* strain actually reduces overall virulence.

In summary, our data indicate that Plb1 plays a key role both in intracellular survival within host phagocytes and in driving cryptococcal cell size changes both *in vitro* and *in vivo*.

## Supplementary Material

Supplemental material
