# Supplementary material for: Cryptococcal Phospholipase B1 Is Required for Intracellular Proliferation and Control of Titan Cell Morphology during Macrophage Infection
Source: Infect Immun. 2015 Mar 17;83(4):1296–304. doi: 10.1128/IAI.03104-14 (PMC4363446; doi:10.1128/IAI.03104-14)
Supplement: Supplemental material [file supp_83_4_1296__index.html]

Cryptococcal Phospholipase B1 Is Required for Intracellular Proliferation and Control of Titan Cell Morphology during Macrophage Infection — Supplemental material 

# Cryptococcal Phospholipase B1 Is Required for Intracellular Proliferation and Control of Titan Cell Morphology during Macrophage Infection

## Supplemental material

**Files in this Data Supplement:**

- Supplemental file 1 -

  Fig. S1. (Ai) Total burden of infection within a fixed population of J774 cells following 2 h of incubation with anti-capsule antibody-opsonized cryptococci at an MOI of 1:10. (Aii) Mean intracellular proliferation rate for anti-capsule antibody-opsonized H99, Δ*plb1*, and Δ*plb1*::*PLB1* cells within murine J774 macrophages. (Bi) Total burden of infection within a fixed population of J774 cells following 2 h of incubation with opsonized cryptococci at an MOI of 1:10. (Bii) Mean intracellular proliferation rate for pooled human serum-opsonized H99, Δ*plb1*, and Δ*plb1*::*PLB1* cells within murine J774 macrophages. (Ci) Total burden of infection within a fixed population of J774 cells following 2 h of incubation with unopsonized cryptococci at an MOI of 1:10. (Cii) Mean intracellular proliferation rate for unopsonized H99, Δ*plb1*, and Δ*plb1*::*PLB1* cells within murine J774 macrophages. Table S1. Mann-Whitney U test statistical analysis for cell size experiments inside J774 macrophages as shown in Fig. 4. Table S2. Mann-Whitney U test statistical analysis for cell size experiments in DMEM alone as shown in Fig. 4.

  PDF, 169K
