# Supplementary material for: Cryptococcal Phospholipase B1 Is Required for Intracellular Proliferation and Control of Titan Cell Morphology during Macrophage Infection
Source: Infect Immun. 2015 Mar 17;83(4):1296–304. doi: 10.1128/IAI.03104-14 (PMC4363446; doi:10.1128/IAI.03104-14)
Supplement: Supplemental material [file IAI.03104-14_zii004151143so1.pdf]

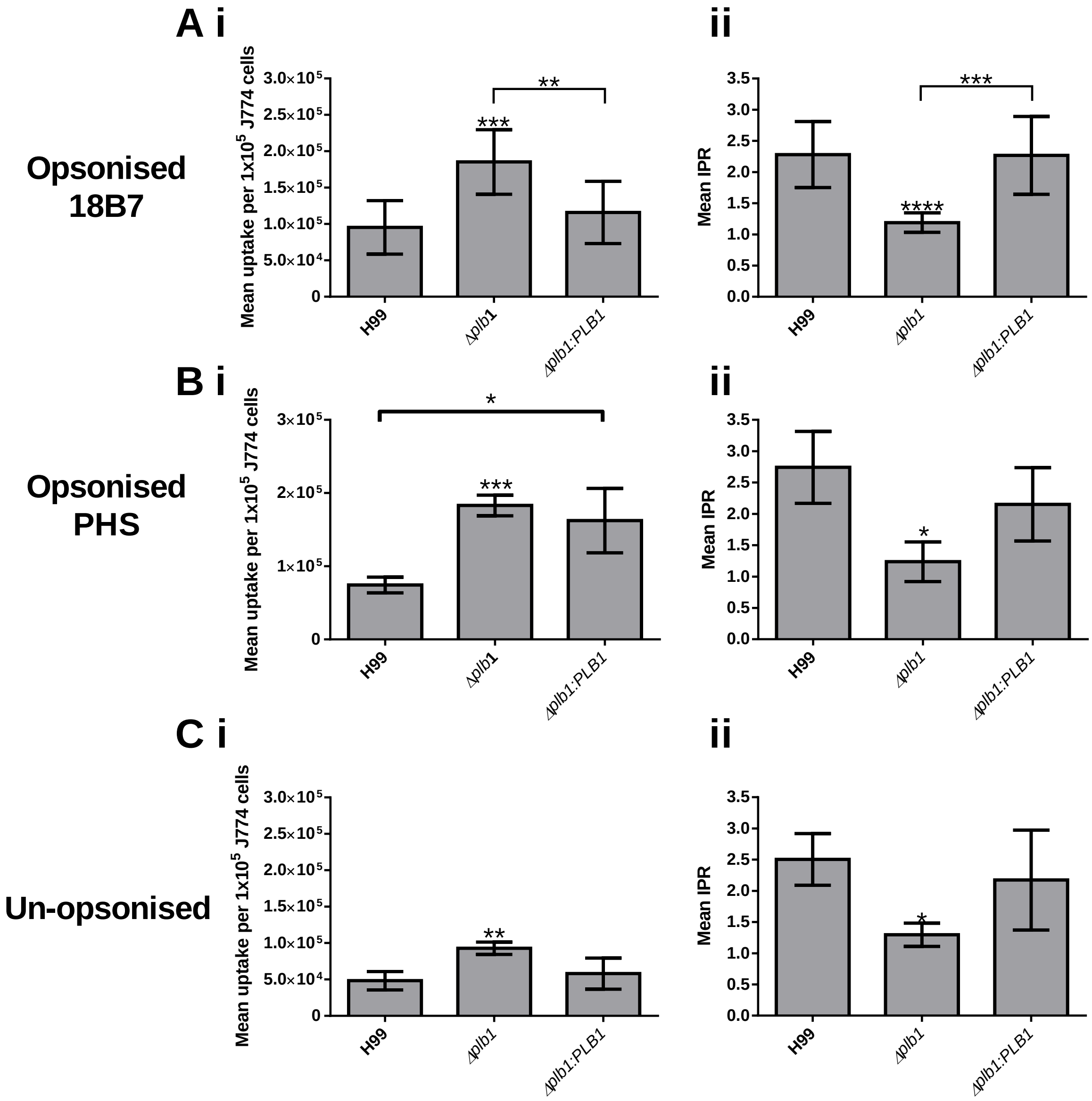
A i
ii
Opsonised
18B7
Mean uptake per 1x10^5 J774 cells
3.0×10^5
2.5×10^5
2.0×10^5
1.5×10^5
1.0×10^5
5.0×10^4
0
**
***
Mean IPR
3.5
3.0
2.5
2.0
1.5
1.0
0.5
0.0
***
****
H99
Δplb1
Δplb1:PLB1
B i
ii
Opsonised
PHS
3×10^5
2×10^5
1×10^5
*
***
C i
ii
Un-opsonised
**

Supplementary figure 1 – Note – Supplementary figure 1 Ai is reproduced from Figure 1 B; it has been reproduced for ease of comparison. **A i** Total burden of infection within a fixed population of J774 cells (1x10^5) following 2 hours incubation with anti-capsule antibody (18B7) opsonised cryptococci at a MOI of 1:10. N=9. *** H99 vs. *Δplb1 p = 0.0003.* ** *Δplb1 vs Δplb1:PLB1* p = 0.0038 (Two tailed unpaired T test). **A ii** Mean intracellular proliferation rate for anti-capsule antibody (18B7) opsonised H99, *Δplb1* and *Δplb1:PLB1* within murine J774 macrophages. N= 8. **** H99 vs. *Δplb1* p= <0.0001, *** *Δplb1* vs. *Δplb1*:PLB1 p= 0.0003 (Two tailed unpaired T test). **B i** Total burden of infection within a fixed population of J774 cells (1x10^5) following 2 hours incubation with opsonised cryptococci at a MOI of 1:10. N = 3. *** H99 vs. *Δplb1* p = 0.0004 (Two tailed unpaired T test). * H99 vs. *Δplb1:PLB1* p = 0.028.**B ii** Mean intracellular proliferation rate for pooled human serum opsonised H99, *Δplb1* and *Δplb1:PLB1* within murine J774 macrophages. N= 3. (Two tailed unpaired T test). **C i** Total burden of infection within a fixed population of J774 cells (1x10^5) following 2 hours incubation with unopsonised cryptococci at a MOI of 1:10. N=3. ** H99 vs. *Δplb1* p = 0.0073 (Two tailed unpaired T test). **C ii** Mean intracellular proliferation rate for unopsonised H99, *Δplb1* and *Δplb1:PLB1* within murine J774 macrophages. N=3. * H99 vs. *Δplb1* p= 0.0101 (Two tailed unpaired T test).

Measurement	Comparison	P value	Mann-Whitney U	Difference between medians (actual)
Cell body diameter (Figure 3A)	H99 0hr vs. H99 18hr	0.0001	1.014e+006	-0.2028
	Δplb1 0hr vs. *Δplb1 18hr*	< 0.0001	546491	1.208
	Δplb1:PLB1 0hr vs. *Δplb1:PLB1* 18hr	< 0.0001	1.956e+006	-0.5025
Capsule thickness (Figure 3B)	H99 0hr vs. H99 18hr	< 0.0001	11394	1.280
	Δplb1 0hr vs. *Δplb1 18hr*	< 0.0001	3745	3.959
	Δplb1:PLB1 0hr vs. *Δplb1:PLB1* 18hr	< 0.0001	10367	1.371
Total diameter (Figure 3C)	H99 0hr vs. H99 18hr	< 0.0001	10680	0.8187
	Δplb1 0hr vs. *Δplb1 18hr*	< 0.0001	1216	7.275
	Δplb1:PLB1 0hr vs. *Δplb1:PLB1* 18hr	< 0.0001	11269	2.156
Ratio cell body to capsule (Figure 3D)	H99 0hr vs. H99 18hr	< 0.0001	10608	0.1257
	Δplb1 0hr vs. *Δplb1 18hr*	< 0.0001	2504	0.5094
	Δplb1:PLB1 0hr vs. *Δplb1:PLB1* 18hr	0.6907	19736	0.008603

Supplementary Table 1 – Mann Whitney U test Statistical analysis for cell size experiments inside J774 macrophages as shown in figure 4.

**Measurement**	**Comparison**	**P value**	**Mann-Whitney U**	**Difference between medians (actual)**
Cell body diameter (Figure 3A)	H99 0hr vs. H99 18hr	< 0.0001	132678	-0.8300
	Δplb1 0hr vs. *Δplb1 18hr*	< 0.0001	165572	-0.6700
	Δplb1:PLB1 0hr vs. *Δplb1:PLB1* 18hr	< 0.0001	159019	-0.4900
Capsule thickness (Figure 3B)	H99 0hr vs. H99 18hr	< 0.0001	7343	2.340
	Δplb1 0hr vs. *Δplb1 18hr*	< 0.0001	9941	2.330
	Δplb1:PLB1 0hr vs. *Δplb1:PLB1* 18hr	< 0.0001	8137	1.500
Total diameter (Figure 3C)	H99 0hr vs. H99 18hr	< 0.0001	12835	3.410
	Δplb1 0hr vs. *Δplb1 18hr*	< 0.0001	11712	2.975
	Δplb1:PLB1 0hr vs. *Δplb1:PLB1* 18hr	< 0.0001	14594	2.200
Ratio cell body to capsule. (Figure 3D)	H99 0hr vs. H99 18hr	0.0675	41824	-0.01000
	Δplb1 0hr vs. *Δplb1 18hr*	< 0.0001	13214	0.3750
	Δplb1:PLB1 0hr vs. *Δplb1:PLB1* 18hr	< 0.0001	24158	0.0600

Supplementary table 2 - Mann Whitney U test Statistical analysis for cell size experiments in DMEM alone as shown in figure 4.
